# Mycobacterium Celatum, a rare cause of SSTI in the immunocompetent.

**DOI:** 10.51894/001c.144207

**Published:** 2025-09-30

**Authors:** Haris Aleem

**Affiliations:** 1 Internal Medicine Detroit Medical Center-Sinai Grace Hospital

## 59

### CASE

A 48 year old woman with a history significant for dermatomyositis presented with swelling of the right hand extending from ulnar border of hand to volar and dorsal aspects of wrist. Of note, She had undergone multiple steroid injections of her hands in the past for pain control with the most recent one a month before presentation. U/S hand showed widespread right hand and wrist inflammatory tenosynovitis with overlying edema. Possible empyema and phlegmon development was also noted. Patient underwent 3 successful incisions and drainage over the next 4 months for recurrent abscess formation but faced poor healing and reemergence of symptoms after an initial phase of improvement. She was treated with antibiotic regimens consisting of vancomycin, ciprofloxacin, doxycycline, cephalexin and TMP-SMX. Abscess cultures failed to grow any organisms, initially. The chronic nature of the patient’s abscess recurrence raised suspicion for infection with atypical organisms and mycobacterial cultures were sent. Mycobacterium Celatum was isolated from two different abscess cultures 3 months apart. Patient is currently on a course of ethambutol, ciprofloxacin and clarithromycin, to be continued for a year.

### DISCUSSION

Mycobacterium Celatum is an obscure non-tuberculous member of the mycobacterium family. Published data mostly describes widespread pulmonary infections in immunocompromised patients, especially amongst the HIV positive population or colonization in patients with underlying respiratory disease.

Immunocompetent individuals on the other hand are more likely to suffer lymphadenitis or skin and soft tissue infections which are likely to be contracted from direct inoculation raising suspicion that our patient might have acquired it from a previous steroid injection.

Given the rarity of the organism and relative difficulty in culturing it, patient’s are likely to suffer exacerbation of symptoms and worsening of overall prognosis as they might not receive the correct treatment on presentation. Adding to the challenge of diagnosis are limitations of current molecular methods, resource constraints and ongoing development of diagnostic markers.Nonetheless, it is important to consider atypical mycobacteria as an important causative organism amongst patients with therapy resistant chronic SSTis. As evidenced from our case; treatment might require extensive excision of affected tissue along with prolonged susceptibility directed treatment.

